# Association of DNA Damage Repair Gene Polymorphisms *hOGG1*, *XRCC1*and *p53* with Sickle Cell Disease Patients in India

**DOI:** 10.4084/MJHID.2015.046

**Published:** 2015-07-02

**Authors:** Sudhansu Sekhar Nishank

**Affiliations:** Division of Genetics, Regional Medical Research Centre for Tribals (ICMR), Nagpur Road, PO-Garha, Jabalpur-482003, Madhya Pradesh, INDIA

## Abstract

**Background:**

Oxidative stress constitutes one of the significant cause of vaso-occlusive clinical episodes in sickle cell disease (SCD) patients. It brings about the generation of reactive oxygen species and consequent damage to DNA. DNA damage repair genes such as *hOGG1, XRCC1* and *p53* play an important role in the repair of DNA damage during oxidative stress. However, it is not known as to the role of these genes in oxidative stress mediated vaso-occlusive clinical complications of SCD patients.

**Objective:**

To see the possible association of DNA repair gene polymorphisms with clinical manifestation of SCD patients. Methods: Genotyping of DNA damage repair genes by PCR-RFLP, measurement of oxidant and anti-oxidant status, along with a clinical evaluation of 250 SCD patients and their comparison with normal individuals.

**Result:**

The level of oxidants were high, and that of antioxidants were low in SCD patients compared to normal individuals. The prevalence of mutant alleles of *hOGG1* gene, *XRCC1* gene (codon 280 Arg>His) were found to be significantly higher among SCD patients as compared to controls. However, SCD patients did not show clinical association with any of these DNA repair gene polymorphisms.

**Conclusion:**

This indicates that *hOGG1, p53*and *XRCC1* gene polymorphisms have no clinical association with SCD patients in India.

## Introduction

Sickle cell disease (SCD) patients (homozygous sickle cell, Hb SS) are usually associated with activation of enzymatic and non-enzymatic sources of reactive oxygen species (ROS) leading to oxidative stress. The resultant oxidative stress leads to microvascular dysfunction, tissue injury and vaso-occlusive crisis led morbidity and mortality among SCD patients.^1^ In higher eukaryotes, DNA is prone to oxidative damage by ROS, which leads to replication errors and genome instability. This oxidative base damage is highly mutagenic, and if unrepaired it produces life threatening malignancies in human. Sickle cell traits and sickle cell homozygotes in many parts of the world have also been found to be associated with malignancies such as renal medullary carcinoma, multiple myeloma, osteosarcoma, malignant fibrous histiocytoma in the distal tibia.^2^,^3^,^4^ The DNA damage repair genes, such as *hOGG1*(human 8-oxoguanine DNA glycosylase 1) gene, X-ray repair cross complementing group1(*XRCC1*) and tumor suppressor gene*p*53, play important role in repair of DNA damage caused by oxidative stress during DNA replication. Polymorphisms of *hOGG1* Ser>Cys, p53 Arg>Pro and XRCC1 (codon 194 Arg>Trp, codon 280 Arg>His, codon 399 Arg>Gln) have been variably associated with repair of oxidative stress mediated DNA damage in normal human.^5^ Transfused sickle-β-thalassemia patients who did not undergo chelation shows significant damage to DNA due to oxidative stress compared to normal individuals.^6^ Sickle cell disease patients treated with hydroxyurea showed a higher level of DNA damage than controls irrespective of gender, smoking or age.^7^ Oxidative stress promotes vaso-occlusive events and consequent clinical complications in SCD patients. It is known that DNA damage repair genes (enzymes) act as sensitive biomarkers of DNA damage repair efficiency of the cell.^8^ Although oxidative stress that promotes damage to DNA brings about endothelial dysfunction leading to vaso-occlusive complications in SCD patients, it is likely that there is a relationship between DNA repair gene polymorphism and clinical complications in SCD patients. Thus, the present study aims to find out the possible association of DNA repair gene polymorphisms and clinical symptoms of SCD patients belonging particularly to the state of Madhya Pradesh (India).

## Materials and Methods

The study sample involved about 250 SCD patients (homozygous sickle cell, Hb SS) and250 normal individuals (having phenotype Hb AA) from unrelated families belonging to the state of Madhya Pradesh (India). The samples were collected at the sickle cell clinic of Regional Medical Research Centre for Tribals (ICMR), in the the campus of NSB Medical College Jabalpur, Madhya Pradesh (India). The study sample included individuals belonging to Scheduled Caste (SC), Scheduled Tribe (ST) and general population of state of Madhya Pradesh, where both SCD patients and normal individuals were matched with each other with respect the age and ethnicity. Among the study samples, 120 females, 130 males were SCD individuals whereas 118 females and 132 males were normal individuals.

The study was initiated after obtaining written consent of patients/their parents and approval by an ethical committee of RMRCT (ICMR), India and NSB Medical College. The patients having recent or multiple blood transfusions and infection and pregnant women were excluded from the study. Besides the present study was conducted in accordance with ethical standards of Helsinki Declaration. Clinical complications such as chest pain, abdominal pain, bone joint pain, history of blood transfusion, the degree of splenomegaly, frequency of blood transfusion were recorded from the date of onset of disease.

Oxidant and anti-oxidant status of sickle cell disease and normal individuals were assessed by measurement of plasma/serum level of Vitamin C, Vitamin E, 8-OHdG (8-hydroxy-2′-deoxyguanosine), reduced glutathione (GSH), malondialdehyde (MDA), albumin, activity of lactate dehydrogenase (LDH) and albumin using commercially available Elisa kits (Sigma Aldrich, USA; Cell Biolab, USA) and automated biochemical analyzer wherever necessary. Hemoglobin level was measured by an automated blood cell counter (Sysmex Corporation, Japan).

Genotyping of all DNA repair genes gene was performed by PCR - Restriction Fragment Length Polymorphism technique as per published procedure.^9^–^12^ The p53 codon Arg72Pro was genotyped by using primers – 5′ATC TAC AGT CCC CCT TGC CG3′ and 5′ GCA ACT GAC CGT GCA AGT CA3′. A 296 bp PCR product (denaturation at 95^0^C 5 min, 35 cycles of 40 s at 95^0^C, 40 s at 60^0^C and 40 s at 72^0^C) was cleaved by *Bst UI* enzyme (New England Bio Lab) and run in 2% agarose gel.^9^ Homozygous Arg/Arg individuals had two fragments of 169 and 127 bp. Homozygous Pro/Pro individuals had a single fragment of 296 bp, and heterozygous Arg/Pro individuals revealed all three fragments. In case of *XRCC1* gene, Arg to Gln substitution in exon 10 (codon 399) was amplified to form an undigested fragment of 242 bp using primer pairs 5′ CCC CAA GTA CAG CCA GGT 3′ and 5′ TGT CCC GCT CCT CTC AGT AG3′. The PCR product was digested with *Msp1* and analyzed in 2% agarose gel. Homozygous Gln-Gln individuals reflected a single product of 242 bp, homozygous Arg-Arg individuals demonstrated both 148- and 94-bp fragment whereas Arg-Gln individuals revealed all three of the fragments.^9^ For Arg to Trp substitution in exon6 (codon 194 of *XRCC1* gene), a 485 bp PCR fragment was obtained using primer pairs 5′ GCC AGG GCC CCT CCT TCA A3′ and 5′TAC CCT CAG ACC CAC GAG T3′. The PCR products were digested with *PVU II* and analyzed in a 2% agarose gel. Homozygous Arg-Arg individuals reflected a single product fragment of 485 bp whereas homozygous Trp-Trp individuals demonstrated both 396- and 89- bp fragments and heterozygous Arg-Trp individuals revealed all three of fragments.^10^ For *XRCC1* Arg280His codon, a PCR product of 304 bp fragment demonstrates homozygous dominant whereas digestion of PCR product by *Rsa I* into 246 bp- and 58 bp fragments demonstrate homozygous mutant. Primers used for it were 5′CCC CAG TGG TGC TAA CCT AA3′ and 5′CTA CAT GAG GTG CGT GCT GT3′.^11^ For *XRCC1* gene, PCR conditions were denaturation at 95^0^C 5 min, 35 cycles of 40 s at 95^0^C, 40 s at 60^0^C and 40 s at 72^0^C). Genotyping of *hOGG1* Ser 326 Cys was done by PCR-RFLP involving primers – 5′TTG CCT TCG GCC CTG TTC CCC AAG GA3′,5′ TTG CTG GTG GCT CCT GAG CAT GGC CG3′ and restriction enzyme *Msp I*. The PCR product was 168 bp and *MspI* digested products were 142, 26 bp for homozygous mutant. The PCR condition was same as described for *XRCC1* gene except the annealing temperature kept at 65.5^0^C.^12^

Statistical analysis for *Fisher*’s exact *X*^2^ test using the odds ratio (OR), 95% con dence interval were performed by statistical software Graph Pad Prism version 5.0 (La Jolla, CA, USA). The *X*^2^ test was also used to test the Hardy-Weinberg equilibrium among the study subjects. The value of *P* < 0.05 was considered to be significant.

## Results

The mean age of SCD patients was 16.3 (±6.0, Standard Deviation) years, and that of the control group was 17.6 (±6.8, SD) years. The frequency of mutant alleles *hOGG1* 326Cys (51.8%) and *XRCC1* 280 His (54.8%) were found to be significantly high in SCD individuals as compared to normal individuals (33.8% and 36.4% respectively). On the other hand there were no significant differences in the frequency of *XRCC1* codon 194 and codon 399 alleles as well as mutant allele of p53 genes (Table 1 and Table 2) between SCD and normal individuals. Comparison of oxidant and antioxidant status of SCD and normal individuals showed that SCD patients had significantly lower level of antioxidants such as Vitamin C, Vitamin E, albumin as compared to controls. On the other hand, SCD individuals had significantly higher level of oxidants such as 8-OHdG and MDA along with a lower level of GSH along with lower activities of LDH (Table 3). There were no difference among the SCD patients with and without mutations in *hOGG1* 326Cys and *XRCC1* 280His alleles with respect to yearly incidence of chest pain, bone joint pain, fatigue, fever, blood transfusion along with degree of splenomegaly and age onset of disease (Table 4).

## Discussion

Clinical complications of sickle cell disease (SCD) involve generation and impairment of oxidative stress. There is a relationship between markers of oxidative stress and common secondary diseases in SCD such as acute chest syndrome and pulmonary hypertension. Autoxidation of sickle hemoglobin (HbS) along with repeated cycle of sickling and unsickling cause premature destruction of erythrocytes and generation of reactive oxygen species (ROS) leading to oxidative stress.^1^ This is evidenced in the present study which shows low level of antioxidants (such as Vit C, Vit E, albumin, GSH) and high level of oxidants (such as 8OHdG, MDA) in SCD patients compared to normal individuals. The lower level of antioxidants particularly Vit C, Vit E, albumin, GSH along with higher level of LDH, low level of hemoglobin in this study are supported by earlier findings in SCD patients.^13^ The high level of MDA in SCD patients observed in the present study is similar to earlier observation of higher level of MDA produced in related hemoglobinopathy patients particularly beta thalassemia patients of India.^14^ Oxidative products such as MDA and 8-OHdG are known to be mutagenic and cause damage to DNA. Besides low levels of antioxidants such as Vitamin E and Vitamin C are found to cause genome instability and damage DNA.^15^ Both of these events may promote carcinogenesis if these oxidants accumulate in the cells. However, DNA repair genes play a significant role in the repair of DNA damage caused by oxidants. It is observed that combination of different variants in DNA damage repair enzymes may modulate the production of 8-oxoguanosine adducts in white blood cell exposed to mutagens.^16^ Similarly individuals carrying *XRCC1* 280 His allele and *hOGG1* 326Cys allele have an increased risk of chromosomal aberrations and many individuals having these mutant alleles have higher incidence of malignancy in normal population.^17^,^18^,^19^ Although the frequency of these mutant alleles of *XRCC1* and *hOGG1* genes is found to be elevated in a present study of SCD patients; these alleles seem incapable of explaining the usual clinical complications of SCD patients. Rather, adhesion of the sickle cell to endothelium due to oxidative stress cause inflammation that leads to the major cause of clinical symptoms in SCD. 13

Therefore, it appears that there is absence of association between DNA repair gene polymorphisms and clinical symptoms, as reflected in the present study. Frequency of *XRCC1* 280 His allele in normal individuals of the present study is different from the normal population in Asian (7 to 15%), African (3%), Caucasians (5 to 9%). Similarly, frequency of *hOGG1* 326 Cys allele is different from other countries (39 to 74% in China, 20.2% in Caucasians) including earlier Indian study (28 to 29%).^20^,^21^ This discrepancy in findings may imply variation in ethnicity, environmental factors.

However, further study of SCD populations from different communities of India may give insights on the role of DNA damage repair gene polymorphisms in clinical manifestation of SCD patients. Thus given the present study findings, it may be concluded that *hOGG1*, *XRCC1*, and *p53* gene polymorphisms do not seem to play a significant role in clinical manifestations of SCD patients of India.

## Conclusion

There is no significant differences in the distribution and clinical impact of *hOGG1, p53*and *XRCC1* gene polymorphisms among SCD patients in India.

## Figures and Tables

**Table 1:** Genotype and allele frequencies of *hOGG1* codon 326 Ser>Cys and *p53* 72Arg>Pro polymorphisms among SCD patients and control group.

Genotype/Alleles	SCD patient n=250	Control group n=250	Odds ratio # 95% confidence interval	*P* value#
*hOGG1* codon 326 Ser> Cys				
Ser/Ser	60 (24.0%)	123 (49.2%)	0.32 (0.22 – 0.47)	< 0.0001
Ser/Cys	121 (48.4%)	85 (34.0%)	1.82 (1.27 – 2.61)	0.001
Cys/Cys	69 (27.6%)	42 (16.8%)	1.88 (1.22 – 2.9)	0.005
Alleles331				
Ser (Wild)	241 (48.2)	331 (66.2)		
Cys (Mutant)	259 (51.8)	169 (33.8)	2.1 (1.63 – 2.71)	< 0.0001
*p53* codon 72 Arg>Pro				
Arg/Arg	83 (33.2%)	98 (39.2%)	0.77 (0.53 – 1.11)	0.192
Arg/Pro	86 (34.4%)	76 (30.4%)	1.2 (0.82 – 1.74)	0.38
Pro/Pro	81 (32.4%)	76 (30.4%)	1.1 (0.75 – 1.6)	0.7
Alleles				
Arg(Wild)	252 (50.4)	272 (54.4)		
Pro(Mutant)	248 (49.6)	228 (45.6)	1.27 (0.99 – 1.63)	0.06

# by Fisher’s exact test two tailed, sample frequency expressed as no.(%).

**Table 2:** Allele and genotype frequencies of *XRCC1* gene polymorphism among SCD patients and controls.

Genotype/Alleles	SCD patients n=250	Control group n=250	Odds ratio# 95% confidence interval	*P* value#
*XRCC1* codon 280 Arg>His				
Arg/Arg	58 (23.2%)	120 (48.0%)	0.32 (0.22 – 0.48)	< 0.0001
Arg/His	110 (44.0%)	78 (31.2%)	1.73 (1.2 – 2.49)	0.004
His/His	82 (32.8%)	52 (20.8%)	1.85 (1.24 – 2.78)	0.003
Alleles				
Arg (Wild)	226 (45.2)	318 (63.6)		
His (Mutant)	274 (54.8)	182 (36.4)	2.11 (1.64 – 2.72)	< 0.0001
*XRCC1* codon 399 Arg>Gln				
Atg/Arg	96 (38.4%)	105 (42.0%)	0.86 (0.6 – 1.23)	0.465
Arg/Gln	119 (47.6%)	113 (45.2%)	1.1 (0.77 – 1.56)	0.653
Gln/Gln	35(14.0%)	32 (12.8%)	1.1 (0.66 – 1.85)	0.793
Alleles				
Arg (Wild)	311 (62.2)	323 (64.6)		
Gln (Mutant)	189 (37.8)	177 (35.4)	0.99 (0.77 – 1.28)	1.0
*XRCC1* codon 194 Arg> Trp				
Arg/Arg	151(60.4%)	162 (64.8%)	0.82 (0.57 – 1.19)	0.355
Arg/Trp	80(32.0%)	74 (29.6%)	1.11 (0.76 – 1.63)	0.628
Trp/Trp	19 (7.6%)	14 (5.6%)	1.38 (0.67 – 2.83)	0.471
Alleles				
Arg (Wild)	382 (76.4)	398 (79.6)		
Trp (Mutant)	118 (23.6)	102 (20.4)	1.20 (0.89– 1.62)	0.252

# by Fisher’s exact test two tailed, sample frequency expressed as no.(%)

**Table 3:** Level of oxidants, anti-oxidants, hemoglobin and LDH in Sickle cell disease patients compared to normal individuals.

	SCD patient (n= 80)	Normal (n= 87)	*P* value#
Vitamin C (mg/dL)	12.56 ± 3.9*	14.62 ± 1.84	< 0.0001
Vitamin E (mg/L)	11.21 ± 3.42	13.04 ± 1.64	< 0.0001
8-OHdG (ng/ml)	0.641 ± 0.117	0.414 ± 0.085	< 0.0001
GSH (mg/1ml packed RBC)	7.27 ± 1.08	6.38 ± 0.96	< 0.0001
MDA (nmol/ml of packed RBC)	617.9 ± 100.0	501.9 ± 44.55	< 0.0001
Albumin (g/dl)	2.707 ± 0.461	3.855 ± 0.78	< 0.0001
LDH (IU/L)	644.8 ± 169.4	356.3 ± 69.69	< 0.0001
Hemoglobin (g/dl)	8.12 ± 1.79	11.03 ± 1.33	< 0.0001

* values expressed in Mean ± Standard Deviation,

# *P* value < 0.05 is significant

MDA: Malondialdehyde, 8OH-dG: 8-hydroxy deoxyguanosine, GSH- reduced glutathione; LDH- lactate dehydrogenase

**Table 4:** Comparison of Clinical variations between SCD patients with and without mutations in *hOGG1*326 Ser>Cys gene and *XRCC1* 280 Arg>His gene.

Symptoms	hOGG1 mutant ( n = 190 )	hOGG1 wild ( n = 60 )	*P* value#	XRCC1 280 Mutant ( n = 192 )	XRCC1 wild ( n = 58 )	*P* value#

Chest pain	170 (89.4)	50 (83.3)	0.253	140 (72.9)	35 (60.3)	0.073

Bone joint pain	130 (68.4)	40 (66.6)	0.874	120 (62.5)	35 (60.3)	0.76

Abdominal pain	165 (86.8)	56 (93.3)	0.246	105 (54.6)	28 (48.2)	0.453

Fatigue	95 (50)	25 (41.6)	0.3	100 (52.1)	31 (53.4)	0.881

Fever (no. of times/yr)						
0 – 5 times	35 (18.4)	8 (13.3)	0.435	27 (14.1)	3 (5.2)	0.103
6 – 12	20 (10.5)	5 (8.3)	0.806	18 (9.3)	9 (15.5)	0.226
> 12	11 (5.7)	3 (5.0)	1.0	8 (4.1)	5 (8.6)	0.186

Splenomegaly						
0 – 2cm	65 (34.2)	27 (45.0)	0.166	77 (40.1)	30 (51.7)	0.131
2 – 4 cm	37 (19.4)	15 (25.0)	0.365	45 (23.4)	20 (34.4)	0.123
> 6 cm	24 (12.6)	10 (16.6)	0.516	18 (9.4)	4 (6.9)	0.791

Blood transfusion frequency(no./yr)						
0 – 3	42 (22.1)	20 (33.3)	0.08	50 (26.0)	20 (34.4)	0.24
4 – 6	28 (14.7)	11 (18.3)	0.54	25 (13.0)	10 (17.2)	0.39
7 – 10	10 (5.2)	6 (10.0)	0.22	10 (5.2)	5 (8.6)	0.34

Age of onset of disease						
0 – 3 yr	15 (7.8)	8 (13.3)	0.2	10 (5.2)	4 (6.9)	0.74
3 – 6 yr	27 (14.2)	14 (23.3)	0.11	12 (6.2)	4 (6.9)	0.76
6 – 9 yr	31 (16.3)	16 (26.6)	0.08	40 (20.8)	18 (31.0)	0.11
> 9 yr	48 (25.2)	22 (36.6)	0.09	60 (31.2)	25 (43.1)	0.11

# by Fisher’s exact test two tailed, sample frequency expressed as no.(%)
